# Perceived Discrimination, Social Environment, and Older Adults’ Psychological Well-Being

**DOI:** 10.1093/geroni/igab046.1780

**Published:** 2021-12-17

**Authors:** Min-Kyoung Park, Christine Mair

**Affiliations:** 1 University of Maryland, Baltimore & Baltimore County, Ellicott City, Maryland, United States; 2 University of Maryland, Baltimore County (UMBC), Baltimore, Maryland, United States

## Abstract

Experiencing discrimination can have detrimental effects on psychological well-being. For older adults in the U.S., discrimination on the basis of country of origin may be a particularly alienating experience. A positive social environment, however, has been shown to buffer associations between discrimination and poorer psychological well-being. However, this hypothesis has not been tested in a sample of older Americans who perceive discrimination because of country of origin. As the United States continues to diversify and politically polarize, understanding older adults’ experiences with discrimination and identifying potential buffers to these negative effects is increasingly important. We analyze 942 older Americans (aged 50+) from the Psychosocial Module of the most recent wave of the Health and Retirement Study (HRS, 2020). Specifically, we analyze associations between perceived discrimination on the basis of country of origin and three psychological well-being outcomes: loneliness, anxiety, and life satisfaction. We further test if the social environment buffers negative effects by examining interactions between discrimination and social support as well as discrimination and neighborhood environment. Our results reveal clear and consistent associations between older adults’ perceived discrimination and increased loneliness and decreased life satisfaction. These negative associations, however, appear to be buffered by social support and positive neighborhood environment, respectively. The potential buffering effect of positive social environments on psychological well-being is particularly pronounced for older adults under the age of 65. We discuss these findings in light of the prevalence of discrimination in the U.S. and consider potential mechanisms for improving the social environment of older adults.

